# Gas6/TAM Signalling Negatively Regulates Inflammatory Induction of GM-CSF in Mouse Brain Microglia

**DOI:** 10.3390/cells10123281

**Published:** 2021-11-24

**Authors:** Shannon E. Gilchrist, Grace M. Pennelli, Sassan Hafizi

**Affiliations:** School of Pharmacy and Biomedical Sciences, University of Portsmouth, Portsmouth PO1 2DT, UK; shannon.gilchrist@port.ac.uk (S.E.G.); Grace.Pennelli@myport.ac.uk (G.M.P.)

**Keywords:** microglia, astrocytes, glial cells, GM-CSF, Gas6, TAM receptors, NF-κB, neuroinflammation, lipopolysaccharide, primary culture

## Abstract

Microglia and astrocytes are the main CNS glial cells responsible for the neuroinflammatory response, where they release a plethora of cytokines into the CNS inflammatory milieu. The TAM (Tyro3, Axl, Mer) receptors and their main ligand Gas6 are regulators of this response, however, the underlying mechanisms remain to be determined. We investigated the ability of Gas6 to modulate the CNS glial inflammatory response to lipopolysaccharide (LPS), a strong pro-inflammatory agent, through a qPCR array that explored Toll-like receptor signalling pathway-associated genes in primary cultured mouse microglia. We identified the *Csf2* gene, encoding granulocyte-macrophage colony-stimulating factor (GM-CSF), as a major Gas6 target gene whose induction by LPS was markedly blunted by Gas6. Both the *Csf2* gene induction and the suppressive effect of Gas6 on this were emulated through measurement of GM-CSF protein release by cells. We found distinct profiles of GM-CSF induction in different glial cell types, with microglia being most responsive during inflammation. Also, Gas6 markedly inhibited the LPS-stimulated nuclear translocation of NF-κB p65 protein in microglia. These results illustrate microglia as a major resident CNS cellular source of GM-CSF as part of the neuroinflammatory response, and that Gas6/TAM signalling inhibits this response through suppression of NF-κB signalling.

## 1. Introduction

Common to many neurodegenerative diseases (such as Alzheimer’s disease or multiple sclerosis), neuroinflammation is often a cause of neuronal damage and eventual cell death [1,2,3]. Microglia and astrocytes work within the CNS to maintain environmental homeostasis and to combat any pathogen- or damage-associated antigens or debris [4,5]. Microglia, the resident immune myeloid cells of the CNS, lead the immune response against these factors through pattern recognition receptors (PRRs) on the cell surface [6]. Through activation of these receptors, microglia are capable of creating a spectrum of distinctive responses that includes enhancing the pro-inflammatory environment [7]. More recently, the role of astrocytes in these responses is being elucidated [8]. Similar to microglia, astrocytes are capable of adopting a pro-inflammatory phenotype in response to external stimuli. This causes a release of pro-inflammatory cytokines, enhancing the environment created by microglia and producing a secondary response [9].

Both microglia and astrocytes express Toll-like receptors (TLRs) on their cell membranes. Microglia express TLRs 1–9 and astrocytes express a more limited selection, with TLRs 2–4 being the main receptors expressed [10,11]. When activated by specific antigens (for example, lipopolysaccharide (LPS) activates TLR4), TLR activation causes a complex signalling cascade via either MyD88-dependent or -independent routes, depending on the TLR [12,13]. Both routes culminate in stimulating intracellular signalling pathways that control transcriptional changes within inflammatory cells, the NF-κB pathway being among the most prominent. Upon inflammatory stimulation, the IκB protein is targeted for proteasome-mediated degradation, allowing the NF-κB protein p65 subunit to translocate to the nucleus and regulate transcription of many pro-inflammatory cytokines and growth factors [14].

The TAM (Tyro3, Axl, Mer) subfamily of receptor tyrosine kinases are key regulators of many biological processes including cell survival and inflammatory resolution [15]. Gas6 is the main ligand of these receptors and vitamin K-dependent γ-carboxylation of its N-terminal domain enables the ligand to activate the receptors [16,17]. Gas6, acting through all three TAM receptors, can alter intracellular signalling pathways associated with inflammation to resolve the inflammatory response within cells, therefore reducing the possibility of chronic inflammatory activation. We and others have previously shown that Gas6, through TAM receptor activation, is vital for inflammatory resolution after TLR-dependent cell stimulation [18,19,20,21,22,23].

The role of Gas6/TAM signalling in the context of the glial component of neuroinflammation remains to be fully elucidated. Here, we studied the effect of Gas6 on microglia and astrocytes under inflammatory stimulation through exploring changes in TLR signalling pathway genes. We identified *Csf2*, encoding the immune regulated growth factor, granulocyte-macrophage-colony stimulating factor (GM-CSF), as a key gene that was suppressed by Gas6 under inflammatory conditions. Furthermore, we discovered that microglia are a major resident CNS cellular source of GM-CSF and that interactions between astrocytes and microglia may alter GM-CSF induction in isolated CNS glial cell systems.

## 2. Materials and Methods

### 2.1. Primary Mouse Glial Cell Cultures

All animal procedures were performed following the Animals (Scientific Procedures) Act, 1986, under a UK Home Office project licence (licence number PC2238199; 22 August 2017) with approval from the institutional ethics committee (AWERB).

Primary glial cells were derived using the protocol from Mecha et al. [24], which was developed from McCarthy and De Vellis who illustrated the purity of the glial cultures [25], and as we have previously described [18,23]. Briefly, forebrains were extracted from neonatal (post-natal day 1–3) wild-type C57Bl/6 mice and were mechanically dissociated in ice-cold DMEM containing 10% foetal bovine serum (FBS; Gibco, Fisher Scientific, Loughborough, UK), 10% horse serum (Gibco, Fisher Scientific) and 1% penicillin/streptomycin (P/S; Gibco, Fisher Scientific; 10:10:1 media) using a serum-coated glass Pasteur pipette. The collected cells were resuspended in warm 10:10:1 media and incubated at 37 °C with 5% CO_2_ for 10–14 days. For experiments using mixed glial cultures, cells were seeded directly onto poly-D-lysine coated 24-well plates after mechanical dissociation. After the 10–14-day incubation period, media was changed to that containing 1% FBS and 1% P/S (1% DMEM) to which treatments were added. To prepare for pure cell cultures, mixed cells were seeded onto sterile poly-D-lysine coated T75 flasks for incubation.

To separate microglial cells, flasks containing mixed glia were orbitally shaken at 230 rpm at 37 °C for 3 h to detach microglia that were then seeded onto poly-D-lysine coated 24-well plates at a cell density of approximately 300,000 cells/well. For astrocyte cells, the original flasks were orbitally shaken for a further 21 h to detach OPCs (not used), leaving a monolayer of astrocytes that were detached and seeded onto a poly-d-lysine coated 24-well plate at a cell density of approximately 200,000 cells/well. Pure cell cultures were allowed to adhere for 1–2 days before the media was changed to 1% DMEM into which treatments were added. Cells were treated with 10 ng/mL of LPS (from *Escherichia coli* O111:B4; Sigma-Aldrich, St. Louis, MO, USA) to induce inflammation. Gas6 ligand pre-treatment (1.6 µg/mL) was added for 1 h before LPS co-incubation for the duration of treatments. The Gas6 concentration used was in the range we have previously determined to be optimally effective for activation of TAM receptors in cells in prior studies [26].

### 2.2. RNA Extraction, Reverse Transcription and qPCR

After experimental treatments, media was removed from wells and cells were lysed for total RNA extraction and purification using the Monarch Total RNA Miniprep Kit (New England Biolabs, Hitchin, UK) according to the manufacturer’s instructions. Briefly, cells were mechanically disrupted from plates and lysed using lysis buffer and RNA purification columns were used to remove contaminants from the extracts through a series of washing and centrifugation steps. Finally, RNA was eluted from the column using nuclease-free water. RNA concentration and purity were measured using a spectrophotometer (ND-1000; NanoDrop Technologies, Wilmington, DE, USA). Equal amounts of total RNA were reverse transcribed (RT) into cDNA (High Capacity cDNA Reverse Transcription Kit, Applied Biosystems, Foster City, CA, USA) which was used to perform quantitative polymerase chain reaction (qPCR) using pre-designed mRNA-specific primer/fluorescent hydrolysis probe sets (Integrated DNA Technologies (IDT), Leuven, Belgium). Gene expression was normalised to *Gapdh* (Thermo Fisher, Waltham, MA, USA) as a reference gene in each sample. Relative gene expression was calculated using 2^−ΔCt^ and fold change in expression was calculated using 2^−ΔΔCt^ depending on the experiment, as previously described [27].

### 2.3. RT^2^ Profiler Toll-Like Receptor Signalling Gene Array

An RT^2^ Profiler PCR Array (Qiagen) was used to explore 84 genes related to TLR signalling. Total RNA was extracted as before, with the addition of a gDNA removal step. The cDNA produced was diluted in PowerTrack SYBR green master mix (Thermo Fisher) and added to the provided 96-well qPCR plate containing primers for each of the genes of interest and a selection of housekeeping genes. Data analysis was conducted using the online Qiagen Gene Globe platform (https://geneglobe.qiagen.com/gb/analyze/ (accessed on 18 August 2021)) which calculated 2^−ΔCt^, fold regulation and fold change values for each gene based on a selection of housekeeping genes included in the array.

### 2.4. Enzyme-Linked Immunosorbent Assay (ELISA)

GM-CSF protein in cell-conditioned media was quantified using the Mouse GM-CSF DuoSet ELISA kit (R&D Systems, UK) following the manufacturer’s instructions. In brief, media and protein standards were added to a Nunc MaxiSorp 96-well plate (Thermo Fisher), initially coated overnight with rat anti-mouse GM-CSF capture antibody and blocked with reagent diluent (1% bovine serum albumin (BSA) in PBS). Biotinylated goat anti-mouse GM-CSF detection antibody was then added, before incubation with streptavidin-HRP reagent. Substrate solution (Thermo Scientific™ Pierce™ TMB Substrate Kit) was then added and the reaction was stopped using 2M H_2_SO_4_. Optical density was measured at 450 and 570 nm (Multiskan GO; Fisher Scientific) for calculation of the GM-CSF protein concentration against a standard dilution series.

### 2.5. Immunofluorescence Staining and Confocal Microscopy

For immunofluorescence experiments, microglial cells were seeded directly onto poly-D-lysine coated glass coverslips (circular, 19-mm diameter; Fisher Scientific) at a cell density of 6 × 10^4^ cells/coverslip. Cells were treated with LPS for 30 min with or without 1 h pre-treatment with Gas6 (1.6 μg/mL). Then media was removed and cells were fixed with 4% paraformaldehyde (Sigma-Aldrich, Merck, Dorset, UK) in PBS for 10 min at room temperature. Coverslips were washed in PBS (3 × 5 min) before fixation with 100% methanol at −20 °C for 10 min and further permeabilisation with 0.3% Triton X-100 at room temperature for 5 min. After a final wash, cells were blocked in 5% horse serum for 1 h at room temperature.

For staining, coverslips were incubated in primary mouse anti-p65 antibody (1:300; Proteintech, Manchester, UK) at 4 °C overnight. Primary antibodies were removed, coverslips were washed as above and AlexaFluor 647 anti-mouse secondary antibody (1:1000; Invitrogen, Fisher Scientific) and DAPI nuclear stain (1:1000; Invitrogen Molecular Probes, Fisher Scientific) were added for 1 h at room temperature. Antibodies and DAPI were diluted in 1% BSA, 0.3% Triton X-100 in PBS. Coverslips underwent a final wash in PBS before being mounted onto clear glass microscopy slides using PermaFluor Aqueous Mounting Medium (Fisher Scientific).

Images were taken on a laser scanning confocal microscope (Zeiss LSM 710, Cambridge, UK) using an oil submersion 63× optical lens. The program RBS *ImageJ* was used for all image processing. Firstly, all images were scaled to micrometres and converted to an 8-bit image for both anti-p65 staining and DAPI staining. A nuclear mask was obtained for DAPI images and the anti-p65 staining intensity for this area was measured (Supplementary Figure S1). The data obtained provided quantitative data for anti-p65 staining within the nuclear area of individual cells.

### 2.6. SDS-PAGE and Western Blotting

For protein extraction, microglia were lysed in ice-cold RIPA buffer (150 mM NaCl, 1% Triton X-100, 0.5% sodium deoxycholate, 0.1% SDS, 50 mM Tris pH 8.0; Fisher Scientific) supplemented with a cocktail of protease and phosphatase inhibitors (Fisher Scientific), incubated at 4 °C for at least 1 h and then mechanically dissociated to fully disrupt cells. Proteins were separated using sodium dodecyl sulphate polyacrylamide gel electrophoresis (SDS-PAGE), using a 10% polyacrylamide gel. After separation, proteins were transferred to an activated polyvinylidene fluoride membrane (Millipore) using semi-dry transfer. After transfer, membranes were blocked for at least 30 min using 3% milk (in TBS-T; Tris-buffered saline and Tween-20), then primary antibodies were added for 1.5 h incubation at room temperature. Membranes were washed in TBS-T before incubation with horseradish peroxidase-conjugated secondary antibodies for 45 min at room temperature. After further washing, membranes were incubated with an enhanced chemiluminescence (ECL) development reagent (Luminata Forte; Millipore) for 3 min in the dark before visualisation with a chemiluminescence CCD camera (ImageQuant LAS 500; GE Healthcare Life Science, Buckinghamshire, UK). *ImageJ* software was used for densitometric quantification of Western blot band intensities [28]. Antibodies used were mouse anti-IκB (1:1000; Cell Signalling Technologies (CST), London, UK), mouse anti-β-actin (1:1000; CST), rabbit anti-cyclophilin (1:1000; Abcam, Cambridge, UK), anti-mouse-HRP (1:5000; Dako, Agilent, Cheadle, UK) and anti-rabbit-HRP (1:10,000; Dako).

### 2.7. Statistical Analysis

Statistical analyses and graphical illustrations were acquired using GraphPad Prism 8. Statistical tests used on appropriate data sets were one-way analysis of variance (ANOVA) or paired Student’s t-tests, whereas non-parametric tests used were Friedman’s tests or Wilcoxon signed-rank tests. A *p* < 0.05 was considered as statistically significant. Data analysed were from a minimum of three independent experiments using separate primary cultures.

## 3. Results

### 3.1. GM-CSF Was Identified as a Novel Target of Gas6 Regulation in Microglia Using a TLR Signalling Gene Array and Confirmed with Gene-Specific qPCR

**Figure 1:**
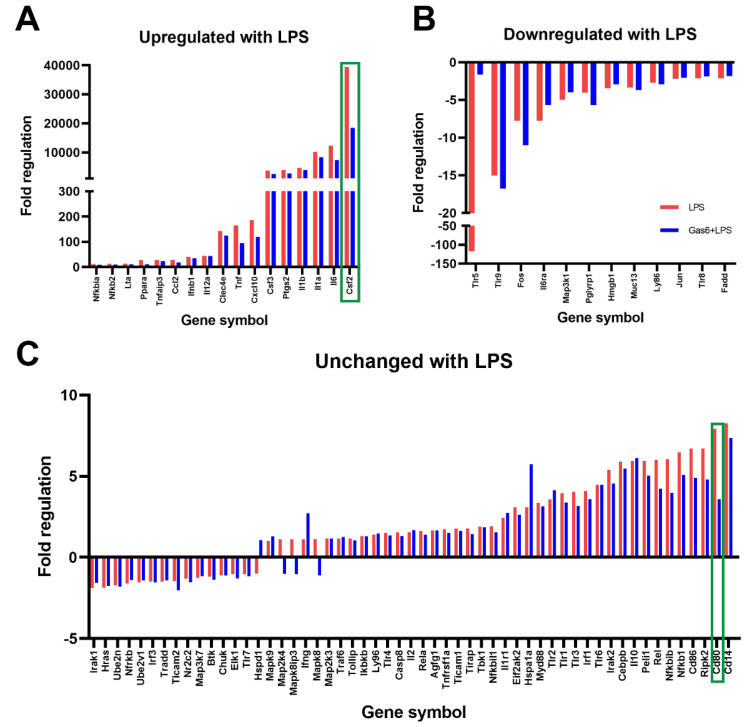
A TLR signalling gene array identified the *Csf2* gene as markedly affected by Gas6/TAM signalling in microglial cells undergoing inflammatory stimulation. An RT^2^ Profiler Gene Array for the TLR signalling pathway was used to measure the expression of 84 genes of interest in pure mouse microglial cultures. Microglia were treated with LPS (10 ng/mL; blue bars) for 8 h in the presence or absence of Gas6 co-incubation (1.6 μg/mL; red bars) added 1 h before and present thereafter. Results are presented as: (**A**) genes upregulated more than 10-fold after LPS stimulation, (**B**) genes downregulated more than 2-fold with LPS or (**C**) genes that were mostly unchanged with LPS treatment. Data is displayed as fold regulation (the negative inverse of fold change for values less than one) of gene expression and is from an exploratory experiment in one mouse culture. Green boxes highlight the genes for which Gas6 presence markedly altered the LPS-induced expression changes.

The RT^2^ Profiler PCR Array was used to identify mouse TLR pathway genes that were regulated by Gas6 in microglial cells under inflammatory conditions. Microglia were treated with LPS (10 ng/mL) for 8 h with or without 1-h pre-treatment with Gas6, remaining present thereafter. Preliminary time course experiments of LPS incubation had shown that 8 h was an optimal time period in which to detect a sizeable change in most genes of interest in these cells. Before use in the gene array, each sample was analysed for *Tnfa* gene expression (not shown) to verify a robust pro-inflammatory response in these cells as we have previously reported [18]. The array results showed a profound gene expression reprogramming response in microglia under the influence of LPS stimulation (Figure 1). Out of all of the genes in the array, two genes, *Csf2* and *Cd80*, were identified for which the presence of Gas6 markedly modified the LPS-induced changes in mRNA expression by over 50%. Moreover, Gas6 co-incubation showed the same suppressive effect on a set of other inflammatory cytokine genes that were upregulated by LPS, including *Il6*, *Il1a*, *Il1b*, *Ptgs2* and *Csf3*, in which Gas6 blunted the LPS effect by at least 15% (Figure 1A). LPS treatment also resulted in downregulation of multiple genes (Figure 1B). Most notable of these was *Tlr5* whose gene expression was downregulated more than 100-fold, an effect that was completely negated by Gas6 presence in this experiment. Most of the remaining genes measured in the array were not altered to a great degree by LPS (>five-fold; Figure 1C). However, we proceeded to analyse *Cd80* further in addition to *Csf2* as it was upregulated more than most other genes in that comparison, and Gas6 blunted the upregulation by approximately 50%.

**Figure 2:**
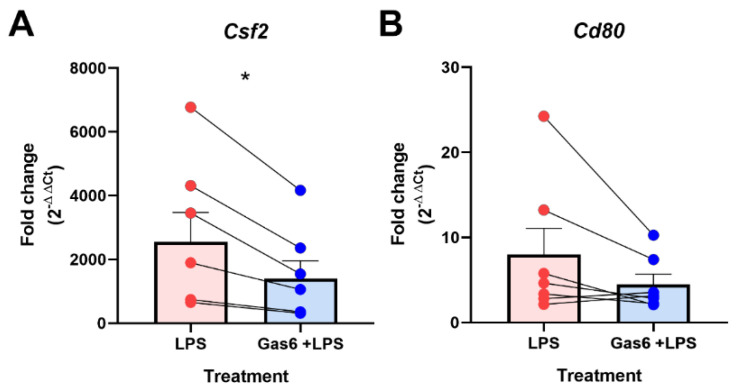
Gas6 inhibits the inflammatory LPS-induced upregulation of *Csf2* mRNA in microglia. RT-qPCR was used to measure the fold change in gene expression of: (**A**) *Csf2* and (**B**) *Cd80* in microglial cultures in response to 8 h LPS (10 ng/mL) exposure in the presence or absence of Gas6 (1.6 μg/mL), which was added 1 h before. Data displayed is the fold change (2^−ΔΔCt^) normalised to non-stimulated cells (value of 1) with each pairwise change illustrated with a connected line for each separate culture and bars displaying mean ± SEM (*n* = 6–7 independent experiments on separate cultures). Data was analysed by Wilcoxon signed-rank test; * *p* < 0.05.

To confirm the PCR array results, gene-specific RT-qPCR was used to measure separately the expression levels of the genes of greatest interest under the same experimental conditions. We also tested Gas6 treatment alone to ensure that it had no basal effects (Supplementary Figure S2). Here we show that, as seen in the array, the *Csf2* gene was profoundly upregulated by LPS stimulation of microglia by several thousand-fold, and co-incubation with Gas6 blunted this induction by approximately 50% (Figure 2A). However, in the analysis of the *Cd80* gene in multiple cultures (*n* = 7), while LPS modestly upregulated the gene in all cultures, we found the Gas6 effect to be variable and therefore overall not significant (Figure 2B). To ensure the observed effects were specific to Gas6-mediated TAM receptor activation, we also used an inactivated form of the Gas6 protein, which did not replicate the clear inhibitory effects observed with the active Gas6 protein (Supplementary Figure S3).

### 3.2. Distinct Profiles of GM-CSF Gene and Protein Induction by LPS in Different Primary Brain Glial Cell Cultures

Granulocyte-macrophage colony-stimulating factor (GM-CSF) is a pro-survival growth factor for myeloid cells that has an important role in inflammation [29]. Thus, due to its massive induction by LPS, and its biological significance in the immune system, GM-CSF was chosen for further exploration in relation to Gas6/TAM signalling. To investigate the time course of *Csf2* induction by LPS, separate primary cultures of pure microglia, pure astrocytes and mixed glia were treated with LPS (10 ng/mL) for 8, 24 and 48 h, and RT-qPCR and ELISA were used to quantify changes in *Csf2* mRNA and GM-CSF protein release, respectively.

**Figure 3:**
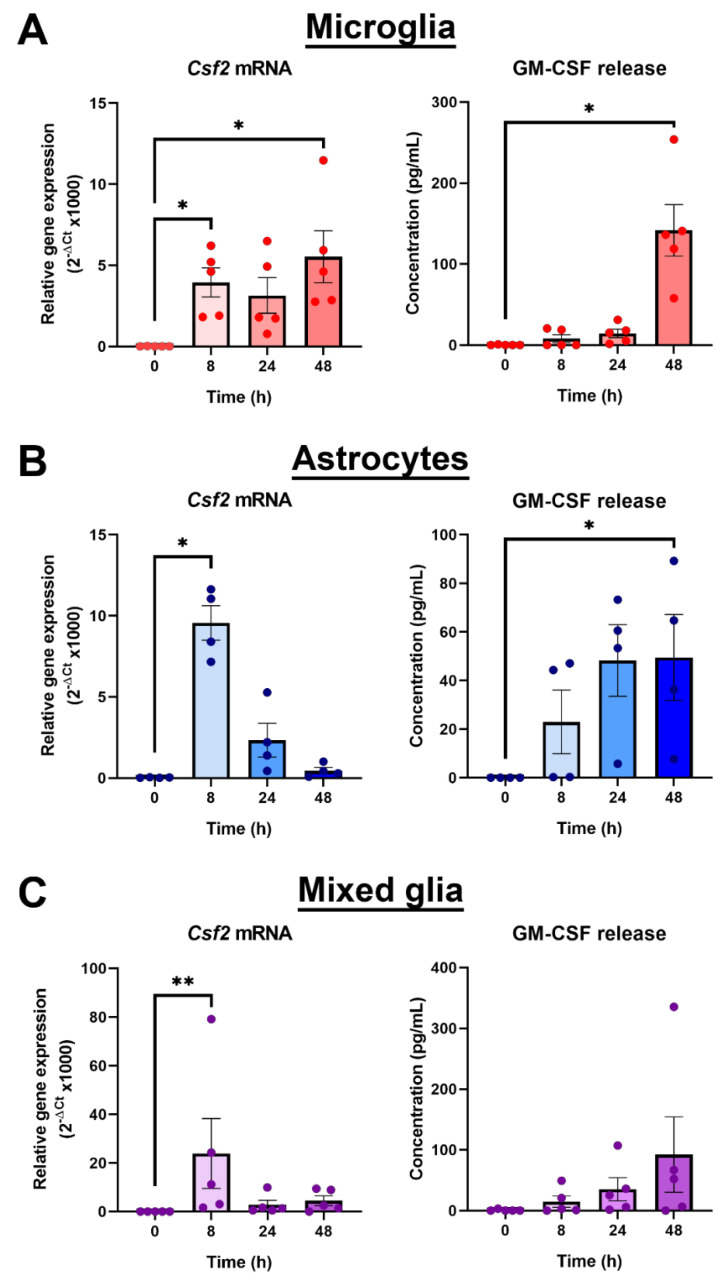
Microglia are the main resident CNS glial source of GM-CSF. Time course of GM-CSF upregulation by LPS (10 ng/mL) in: (**A**) pure microglial cultures, (**B**) pure astrocyte cultures and (**C**) mixed glial cultures, using RT-qPCR and ELISA to measure gene expression and protein release into the cell media, respectively. Gene expression is displayed as relative gene expression (2^−ΔCt^) and protein concentration is in pg/mL. Statistical analysis was determined using Friedman’s tests with *p* < 0.05 (*n* = 4–5 independent experiments on separate cultures). * *p* < 0.05, ** *p* < 0.01.

In microglia, *Csf2* mRNA expression was significantly increased by several thousand-fold after 8 h of LPS incubation, which was sustained for at least 48 h. The shorter time of 1 h with LPS exposure did not stimulate *Csf2* gene expression (data not shown). However, GM-CSF release by cells into the media was not detectable until 24 h after LPS exposure and was significantly increased only at 48 h (Figure 3A). In astrocytes, *Csf2* gene expression was also upregulated by LPS, reaching a peak after 8 h but reverting back to near baseline levels by 24 h (Figure 3B). However, GM-CSF protein release increases were detectable in cultures after 8 h, with significant increases observed after 48 h. Furthermore, the GM-CSF protein concentration measured in the astrocyte culture medium was lower than that of microglial cultures in the majority of cultures compared, to near significance (*p* = 0.0635). Finally, the LPS effect on GM-CSF gene and protein levels was also analysed in primary mixed glial cell cultures, which our own analysis has determined to be mostly composed of astrocytes with approximately 10–15% of microglial cells (Supplementary Figure S4). In the mixed cultures, the time course profile of *Csf2* gene induction by LPS mirrored that of pure astrocyte cultures, whereas a longer period for increased protein detection (at 48 h) was similar to that observed in microglia (Figure 3C).

### 3.3. Gas6 Inhibits LPS-Induced GM-CSF Release in Glial Cultures Containing Microglia

**Figure 4:**
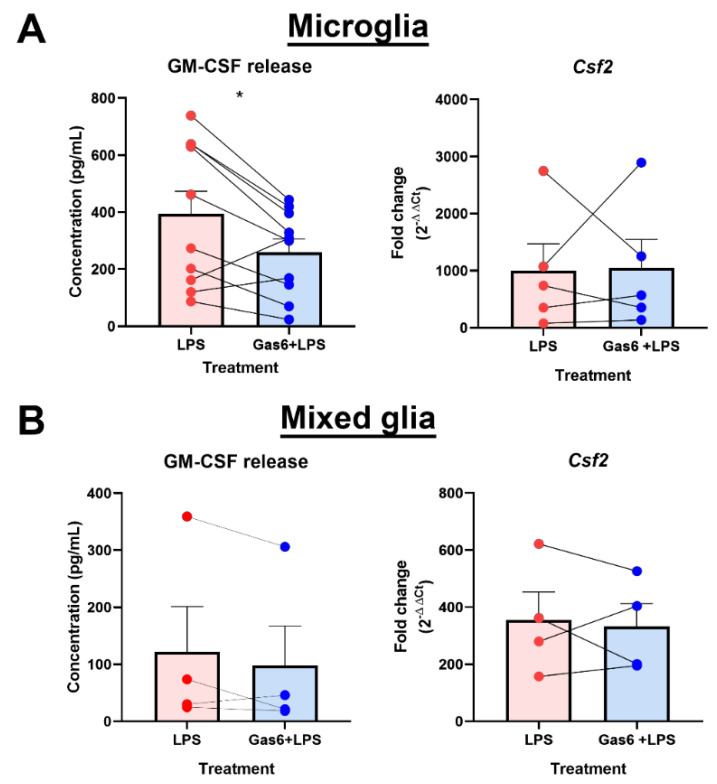
Gas6 supresses the LPS-upregulated GM-CSF protein release by microglia. RT-qPCR and ELISA were used to measure the gene expression and protein release of GM-CSF from: (**A**) pure microglial cultures or (**B**) mixed glial cultures after 48-h LPS treatment (10 ng/mL) with or without 1-h pre-treatment with Gas6 (1.6µg/mL), the Gas6 remaining throughout. Gene expression data shows fold change (2^−ΔΔCt^) and protein release data displays GM-CSF protein concentration (pg/mL). Non-treated microglia had a protein concentration of 0 pg/mL and mixed glia had baseline levels of 0–2 pg/mL (data not shown). Statistical significance was determined using Friedman’s tests for ELISA data or Wilcoxon signed-rank tests for RT-qPCR data with *p* < 0.05 (*n* = 4–10 independent experiments on separate cultures). * *p* < 0.05.

Our initial analysis revealed the LPS-counteracting influence of Gas6 at gene level after 8 h LPS exposure (Figure 2A). However, a 48-h period was required to observe a consistent LPS-induced GM-CSF protein release across all cultures (Figure 3). Therefore, we next determined the effect of Gas6 on GM-CSF protein release following 48 h of exposure to LPS. Microglia and mixed glial cultures were first incubated with Gas6 (1.6 μg/mL) for 1 h prior to addition of LPS (10 ng/mL) for 48 h, with Gas6 remaining throughout. In microglial cultures, GM-CSF levels were significantly decreased with the presence of Gas6, an effect not observed in mixed glia (Figure 4). Furthermore, the decrease observed in microglial GM-CSF protein release was not reflected in *Csf2* gene expression at the same time. Therefore, these results showed that the influence of Gas6 on the microglial inflammatory response was prolonged and sustained over at least a 48-h period.

### 3.4. Gas6 Inhibits Inflammatory NF-κB Pathway Signalling in Microglia

**Figure 5:**
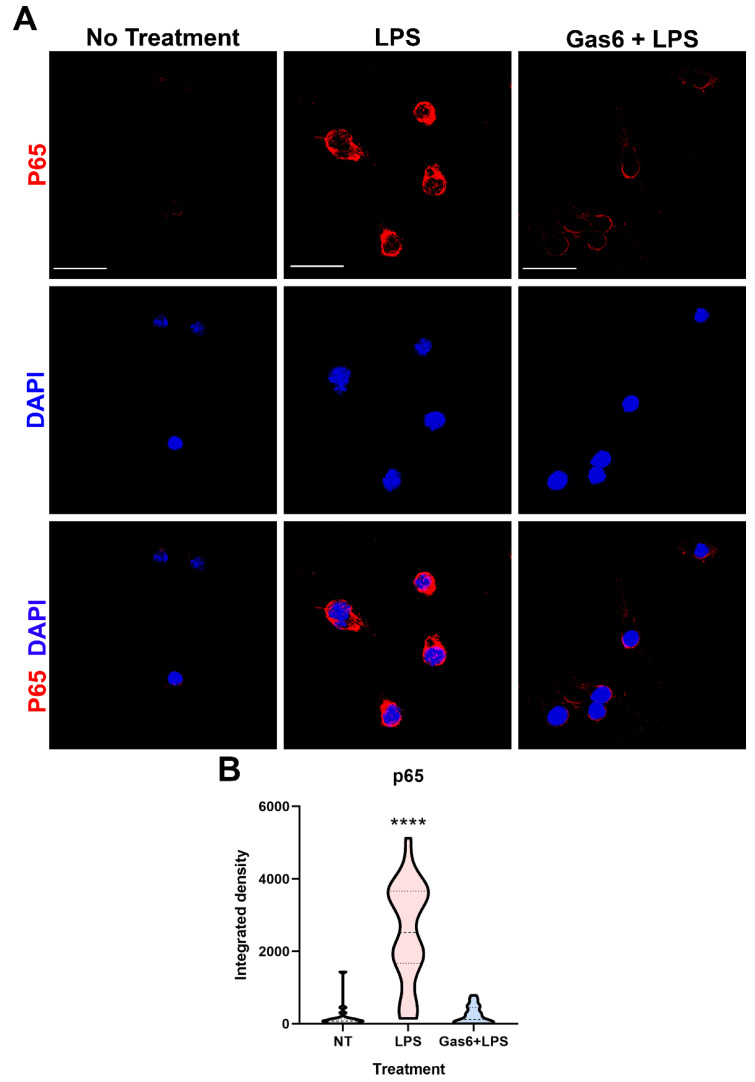
Gas6 inhibits LPS-induced nuclear translocation of the NF-κB p65 subunit in microglia. Pure microglial cell cultures were treated with LPS (10 ng/mL) for 30 min with or without 1 h pre-incubation with Gas6 (1.6 µg/mL), which then remained throughout. (**A**) Cells underwent immunofluorescence staining with anti-p65 primary antibody with AlexaFluor647 anti-mouse secondary antibody and DAPI nuclear counterstaining. Scale bar = 100 µm. (**B**) p65 staining within the nuclear area of each cell was quantified for each treatment group. Data is shown in a violin plot with median and interquartile ranges visible (*n* > 30 cells). Data was statistically analysed using one-way ANOVA; **** *p* < 0.0001 vs. both other conditions.

In order to probe the molecular mechanism by which Gas6 interferes with TLR4 signalling in microglial cells, we investigated the Gas6 effect on pro-inflammatory NF-κB signalling as activated by LPS. Specifically, we monitored the nuclear translocation of the NF-κB p65 subunit following brief exposure to LPS (10 ng/mL) for 30 min, in the presence and absence of Gas6 (1.6 µg/mL), added 1 h before. Immunofluorescence staining and confocal microscopy was used to detect and quantify the intensity of p65 staining inside the nucleus of individual cells. LPS exposure caused a clear and significant increase in the levels of nuclear staining (Figure 5). However, the presence of Gas6 completely inhibited the LPS-induced p65 nuclear translocation. In addition, we investigated the effect of Gas6 on the proteasome-mediated degradation of the IκB protein by western blotting. We observed a clear time course of LPS-induced degradation of IκB protein, with the lowest levels detected at 60 min and levels being partially restored at 90 min (Supplementary Figure S5A). However, in this system, the LPS-induced IκB protein degradation was not observed to be affected by Gas6 (Supplementary Figure S5B).

## 4. Discussion

Microglia, as the major immune cell in the CNS, respond to inflammatory stimuli by altering their phenotype and releasing a plethora of cytokines and soluble factors into the extracellular milieu. Together, this response regulates the course of neuroinflammatory and immune sequelae. In the present study, we report for the first time that the TAM ligand Gas6 is a strong suppressor of the myeloid growth factor GM-CSF in microglia under inflammatory conditions. Our study also identified microglia as a major CNS resident cellular source of GM-CSF, although it was also induced to a lesser degree in astrocytes. We observed a significant upregulation of *Csf2* mRNA expression, peaking at 8 h, and GM-CSF protein release, peaking at 48 h. At both gene and protein levels, the presence of Gas6 markedly inhibited the LPS-stimulated GM-CSF production. Furthermore, Gas6 exerted a profound inhibitory effect on the NF-κB signalling pathway in microglia, as observed through complete inhibition of LPS-stimulated NF-κB p65 nuclear translocation. Thus, our findings demonstrate that Gas6/TAM signalling suppresses microglial GM-CSF production in response to inflammatory stimulation which likely occurs through blockade of NF-κB signalling.

We used a PCR gene array to identify key gene expression changes in pure microglia upon inflammatory stimulation. We also used this to investigate whether Gas6 was able to modulate any of the changes seen. Focussing on the ability of LPS to induce a phenotypic change in microglia, we detected a vast change in the expression of genes associated with TLR signalling, as would be expected in these cells [30]. Although the *Csf2* and *Csf3* genes were included in this array, *Csf1* was not, as it plays an anti-inflammatory role in cells of myeloid lineage whereas the array focused on pro-inflammatory signalling pathway genes [31]. Out of all upregulated genes, the profound increase in *Csf2* gene expression stood out for further investigation. GM-CSF has for many years been known to play a key role in myeloid cell biology [32,33], and its effects on glial cells is also well documented [34,35,36]. In microglia specifically, GM-CSF promotes microglial proliferation and it has been used as an agent to propagate microglial cell cultures in vitro [37,38]. Although many cellular sources of GM-CSF have been documented, including T cells, macrophages and endothelial cells [39], it has not been well established whether microglia themselves are a CNS cellular source of GM-CSF. We demonstrated this in the present study, showing that microglia express high levels of GM-CSF, particularly following inflammatory stimulation. Our findings are supported by earlier indications [40] and also illustrate a capability for microglial autocrine regulation, as has previously been reported [41]. In addition, our results also reveal the potential for some degree of paracrine regulation by astrocytes under the same conditions, as supported by other studies showing that co-incubation of microglia and astrocytes was required for enhanced GM-CSF production [42,43]. Also, GM-CSF has been shown to be important to limit neurodegenerative amyloid accumulation in an Alzheimer’s disease model, further evidence supporting the actions that GM-CSF can have on microglia [40]. Through our investigations using cultures containing both astrocytes and microglia, we demonstrated a gene expression profile similar to that of astrocytes alone, but a protein release profile reflecting that of pure microglial cultures. This suggests that in the mixed cultures, astrocytes (the most numerous cell type in the culture) were the main cells behind the gene expression changes that we observed, however, microglia were the major sources of the GM-CSF protein detected after pro-inflammatory stimulation. Therefore, we have shown that microglia, and astrocytes to a lesser degree, are distinct resident CNS cellular sources of GM-CSF, independent of other immune cells, proving that these glial cells play a role in the inflammatory response in the brain.

Through the PCR array, we also identified two novel target genes that were regulated by co-incubation of Gas6 with LPS: *Csf2* and *Cd80*. Interestingly the array showed that the *Csf3* gene was also upregulated by LPS in microglia; however, Gas6 did not affect this induction as much as it did with the *Csf2* gene. Therefore, Gas6/TAM signalling was specific to regulating *Csf2* gene regulation. In contrast to *Csf2*, the dampening effect of Gas6 on LPS induction of the *Cd80* gene was both smaller and not significant in repeat experiments, suggesting a culture-to-culture variation. CD80 is a membrane bound protein involved in facilitating antigen presentation [44]. Additionally, the array also showed the *Tlr5* gene to be altered by Gas6, however follow-up experiments will need to be done in a future study to explore this further. In the CNS, Gas6 is highly expressed in microglia and present in astrocytes [45,46], and is involved in regulating inflammatory responses within the CNS [15,47]. Generally, Gas6 is able to bind and activate the Tyro3 and Axl receptors on astrocytes, and Mer and Axl on microglia [20,45]. We and others have previously reported the ability of Gas6/TAM signalling to negatively regulate the CNS glial pro-inflammatory response and promote repair. Though use of TAM knockout mice, we observed that Gas6 acts through both Mer and Axl receptors on microglia to suppress TNF-α induction and stimulate pro-repair factors IL-10 and TGF-β [18,23]. Furthermore, after CNS damage, the TAM receptor system is capable of regulating the microglial inflammatory response and promoting the survival of other CNS cells [48]. Here, we have used the same methods as previously reported [18], and have illustrated a profound effect of Gas6 on the *Csf2* gene, with Gas6 halving the LPS gene induction compared to cells exposed to LPS alone. This suppressive effect of Gas6 also translated over a longer period to cellular GM-CSF protein release by microglia exposed to LPS. The similarities observed between our previous work and this study suggest a common pathway for the effects seen on both TNF-α and GM-CSF. Therefore, we illustrate here the ability of Gas6 to strongly suppress yet another immune mechanism with the potential of subduing an aberrant immune response. Considering that GM-CSF can enhance microglial proliferation and promote an inflammatory response, it is important to have a modulatory mechanism in place. Thus, the ability of Gas6 to suppress GM-CSF induction over a prolonged period could provide a novel avenue to therapeutically target GM-CSF in disorders such as multiple sclerosis, where GM-CSF plays a critical role in mediating the disease pathology [49].

As our experiments were conducted entirely on primary microglial cultures from individual mice, this likely gave rise to the variability in degree of gene expression and LPS/Gas6 responses observed. Sex differences could be one factor to account for variability amongst cell cultures as, for example, female rat neonatal microglia have been reported to exhibit less microglial migration, higher phagocytic activity and lower IL-1β in response to LPS compared to male microglia [50]. However, our study was primarily centred on the Gas6 inhibitory effect on LPS inflammatory *Csf2* induction, rather than on the degree of LPS effect itself, and the direction of the Gas6 effect on *Csf2* was largely consistent.

The NF-κB signalling cascade is a multi-step process involving nuclear translocation of the p65 protein and subsequent transcriptional gene changes that lead to upregulation of a variety of pro-inflammatory cytokines and growth factors. TLR-induced inflammatory signalling stimulates the NF-κB pathway in CNS glial cells [14], and furthermore, NF-κB signalling is also involved in the induction of GM-CSF [51]. Therefore, we investigated NF-κB as a potential route for Gas6 to mediate the effects observed on GM-CSF. We observed a strong increase in levels of p65 in the nuclei of microglia exposed to LPS, which was markedly reduced when cells were co-incubated with Gas6. Gas6 has previously been shown to attenuate neuroinflammation through inhibition of the NF-κB family in damaged brain tissue [52] and both the Mer and Axl receptors have been shown to be involved in the Gas6-mediated response [53]. Here, we have expanded the known associations by demonstrating that Gas6 alters GM-CSF signalling through modification of the NF-κB pathway.

In summary, we have shown that, under inflammatory conditions, microglia are a major resident CNS cellular source of GM-CSF, and that Gas6/TAM signalling inhibits this response in addition to others. Furthermore, the Gas6 effect in microglia is mediated through suppression of the NF-κB signalling pathway. Fully untangling the complex inflammatory signalling pathways within the CNS, and the involvement of Gas6 and the TAM receptors in its resolution, could pave the way for novel anti-inflammatory therapies that could benefit patients with inflammatory brain disorders.

## Data Availability

The datasets used and/or analysed during the current study are available from the corresponding author on reasonable request.

## Supplementary Materials

The following are available online at https://www.mdpi.com/article/10.3390/cells10123281/s1, Table S1: Transcript-specific primer-probe sequences for IDT Taqman qPCR primers, Figure S1: Workflow for image analysis of nuclear translocation images, Figure S2: Gas6 treatment alone did not alter the baseline Csf2 expression levels in microglia, Figure S3: Inactivated Gas6 (wGas6) is not effective in reducing Csf2, Figure S4: Mixed glial cultures contain 10–15% of Iba-1-positive cells, Figure S5: Western blot showing IκB protein degradation in microglia exposed to LPS.
