# Supplementary material for: Gas6/TAM Signalling Negatively Regulates Inflammatory Induction of GM-CSF in Mouse Brain Microglia
Source: Cells. 2021 Nov 24;10(12):3281. doi: 10.3390/cells10123281 (PMC8699038; doi:10.3390/cells10123281)
Supplement: Supplementary file 1 [file cells-10-03281-s001.zip › cells-1404500-supplementary.pdf]

Supplementary Table

Supplementary Table S1. Transcript-specific sequences of primers and probes for genesinvestigated in this study.

Gene Symbol	Sequence (5' → 3')	Primers/Probe
Csf2	56-FAM/CGAATATCT/ZEN/TCAGGCGGGTCTGCA/3IABkFQ	Probe
	GTCTCTAACGAGTTCTCCTTCA	Primer 1
	CCTTGAGTTTGGTGAAATTGCC	Primer 2
Cd80	56-FAM/CTGCCTTGC/ZEN/CGTTACAACTCTCCT/3IABkFQ	Probe
	TTGTGCTGCTGATTCGTCTT	Primer 1
	TTGCCAGTAGATTCGGTCTTC	Primer 2

Supplementary Figures

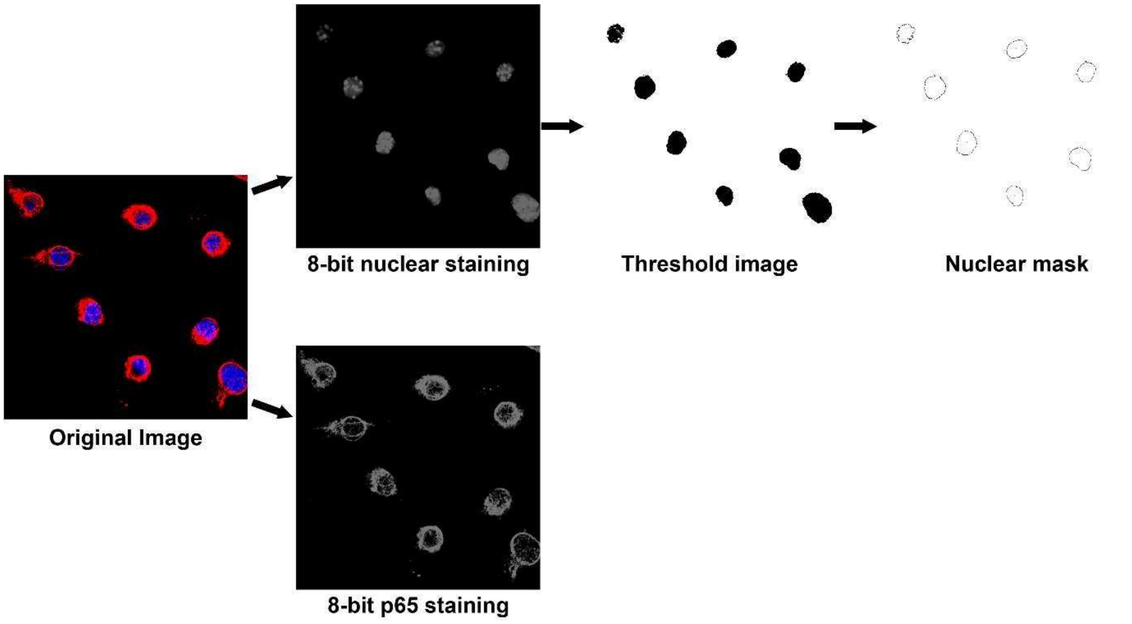

Supplementary Figure S1. Workflow for image analysis of nuclear translocation images. Original images were converted to 8-bit single channel images, with the image of DAPI staining further converted to black and white using 'Threshold' in *ImageJ*. 'Analyse particles' redirected to 8-bit image of p65 was used to measure the Integrated Density of p65 staining within the nuclear mask.

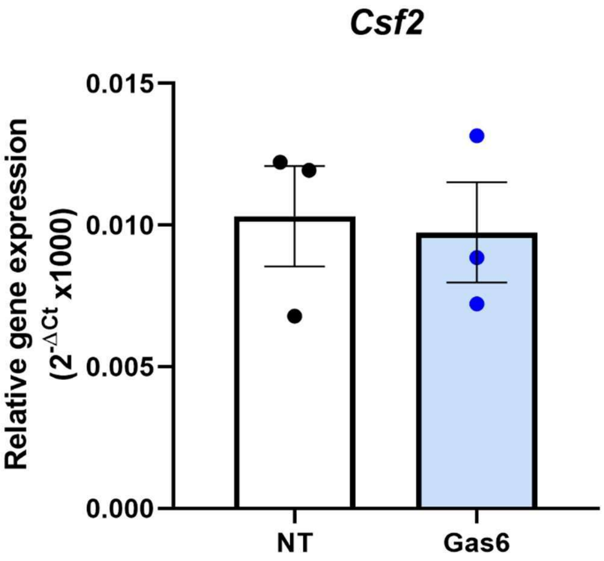

Supplementary Figure S2. Gas6 alone does not alter the baseline Csf2 mRNA expression levels in microglia. RT-qPCR was used to measure the relative gene expression of *Csf2* in microglia exposed to Gas6 for 9h. *Gapdh* was used as reference gene. Statistical analysis was determined using Wilcoxon signed-rank test with $p<0.05$. Data displayed is mean±SEM with individual data points (n=3 experiments on separate cultures).

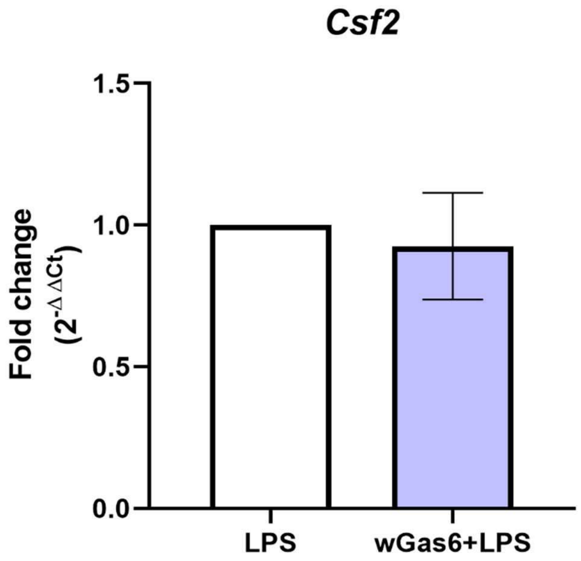

Supplementary Figure S3. Inactivated Gas6 (wGas6) has no effect on LPS-induced *Csf2* geneupregulation in microglia. RT-qPCR was used to measure the fold change in *Csf2* ($2^{-\Delta\Delta Ct}$) in microglia exposed to wGas6 (1.6μg/mL) with LPS (10ng/mL) for 8h (wGas6 added 1h prior to LPS then present throughout), in comparison the LPS alone. *Gapdh* was used as reference gene. Data displayed is mean±SEM with individual data points (n=2 experiments on separate cultures).

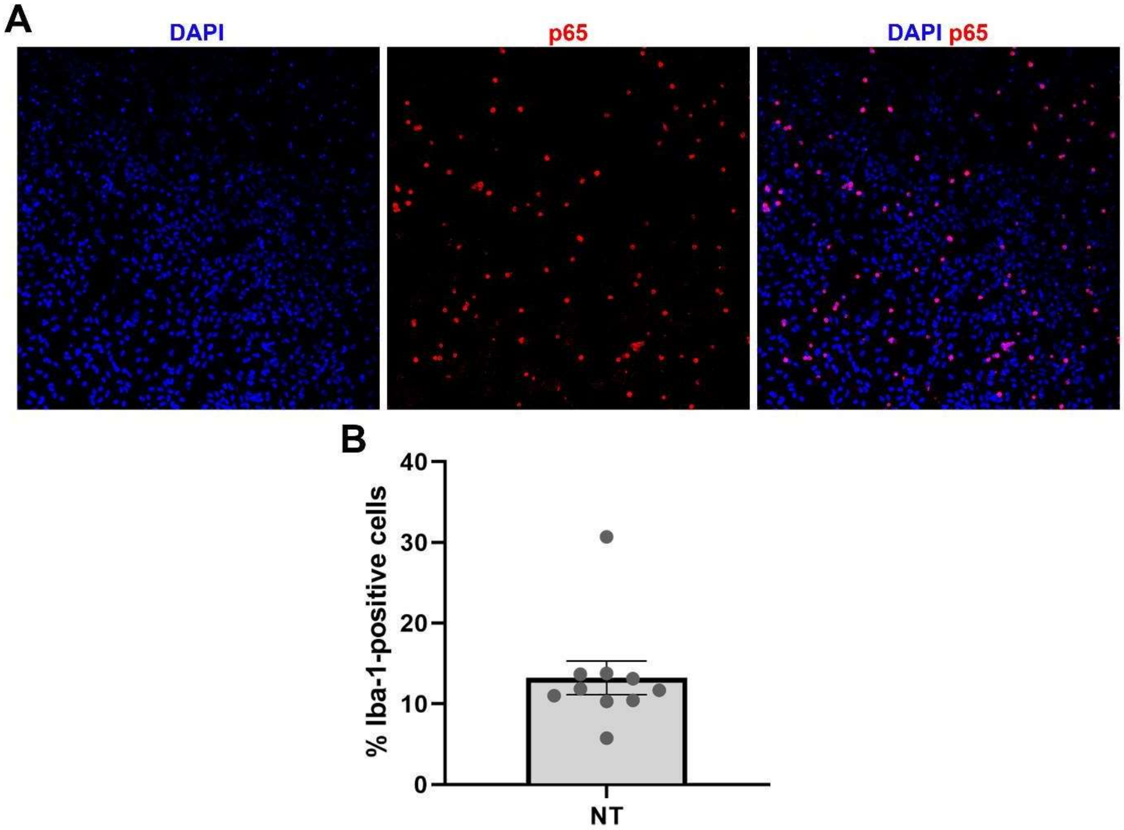

Supplementary Figure S4. Mixed glial cultures contain 10-15% of Iba1-positive cells. (A) Mixed glial cell cultures underwent immunofluorescence staining with Iba1 primary antibody with Cy3 anti-rabbit secondary antibody to stain microglia, and DAPI nuclear counterstaining to stain all cells. (B) Percentage of Iba1-positive cells was quantified with data displayed as mean±SEM with data points displayed from separate images analysed.

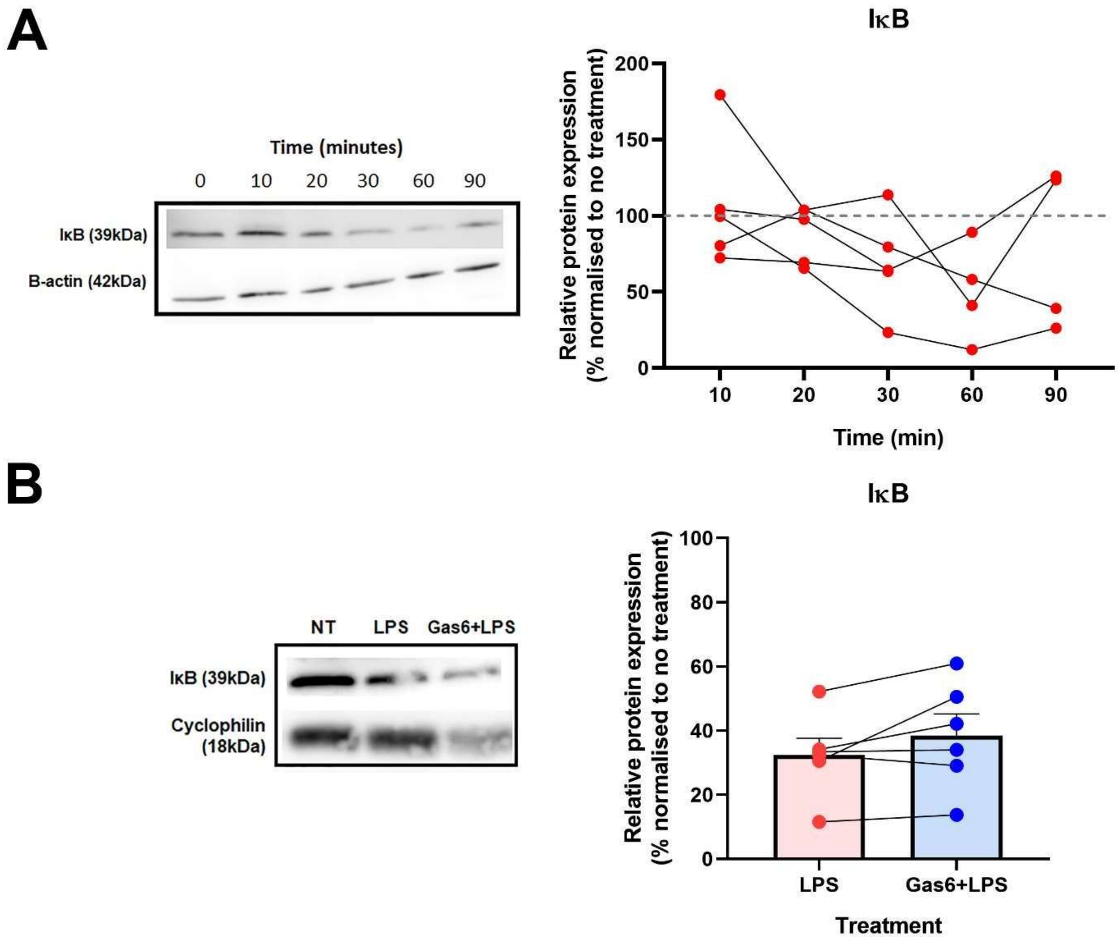

Supplementary Figure S5. Western blot showing IκB protein degradation in microglia exposed to LPS. Microglial cells were treated with LPS (10ng/mL) for (A) a 10-90 min time course or (B) 45 min together with Gas6 (1.6μg/mL), with Gas6 added 1h previously. Protein levels of IκB, as well as loading control proteins β-actin or cyclophilin, were determined by SDS-PAGE and western blotting. Data displayed in bars are from densitometric quantification analysis of IκB western blot band intensities normalised to loading control band intensities. Blot images are representatives of n=4-6 independent experiments using separate cultures. Individual data points from separate experiments are displayed, with lines connecting samples from the same experiment, and in (B) bars also show mean ± SEM relative protein expression.
